# Biography of Living Medical Men*On some of the points here raised by our esteemed correspondent, we shall take the liberty to say a word editorially.—Ed. Buff. Med. Jour.

**Published:** 1856-01

**Authors:** 


### ART. II.—*Biography of Living Medical Men.**

Almost all *great* men, at some period of their mundane existence, ardently desire to embalm the elements of their *greatness*, in unfading ink, on

---

* On some of the points here raised by our esteemed correspondent, we shall take the liberty to say a word editorially.—Ed. Buff. Med. Jour.

the pages of biographical history. Now to what extent this desire should be gratified, is a *grave question*, and, therefore, ought to be referred to *literary undertakers.* But some, less *Barnumized* than others, are too modest to unfold the details of their lives while living, and in the marvelous increase of *great* men, perchance many disappear without leaving for the world a single line to show in what their greatness consisted.

The "Prince of Humbugs," a man who can astonish the world with prodigies, may, with a good show of reason, present, among the rest, his biographical memoir! The "itinerating quack," and vender of patent nostrums, may, with equal propriety, when purchasing whole columns of newspaper-puffs, insert his biographical portrait! Such a brazen exhibition of self-conceit would undoubtedly sell more of his compound, and his patrons, perhaps, would be the better SOLD.

Political demagogues, too, who pull every wire, by hook or by crook, to reach the topmost seat of their ambition, may furnish their *written lives,* accompanied by a lithographic likeness, setting forth the lineaments of greatness in a high forehead, a penetrating eye, and well chiseled features, *(for all these if not possessed can be purchased.)* Such a class of aspirants for riches and worldly renown, invent means best adapted and in keeping with their pursuits.

But may we not discover at the present time, in the medical profession, as it were the copyright of this illegitimate appeal to public favor? If this be true, if medical men while living, and before they have reached the meridian of professional distinction, suffer their lives to be *advertised* in biographical memoir, aught it not to be looked upon with distrust and suspicion, rather than envy? Nay, more! aught it not to be regarded, by every man true to the interests of the profession, as an *epitaph* on *the tombstone* of their professional character?

In these days when the public mind seems more than ever credulous and eager to embrace and cherish the shadow rather than the substance, it is time to draw the line of demarkation between the true character of science and the pretensions of the *Charlatan,* so that it cannot be overlooked or mistaken.

Some there are, who reach a respectable eminence in the profession, and who, for their attainments and contributions to the progress of science, merit the esteem and fraternal regard of professional brethren, but in an hour of temptation, ill fortune and sordid motives, first claim their birth-right; they prove recreant to time-honored principles of professional decorum, and soon find their level among the fraternity of empirics.

If the profession is to be kept unspotted from the stigma of quackery, it must avoid even the appearance of its approval.

Biography of eminent men is of interest to the reader just in proportion to the degree of eminence to which they have attained. This is true of all such writings, but especially does it obtain with regard to the medical profession.

When the toil of life is ended, and the elements of greatness have ceased to develop and unfold on earth, it is time enough to gather and trace those elements from their birth in all their detail. I would not expunge biography from medical literature, but preserve it inviolate, and sacred *to the memory of the* DEAD.

Like the evergreen and myrtle, that mark the spot where the dust returned to the laboratory of earth, so let biographical literature keep ever fresh the memories of the *truly great* and good. The medical profession claims enough, when it takes the body for scientific research; but with shame be it said, that some have even robbed the sepulchre of its literature, flattered by the hope of reaching distinction "by climbing up some other way." They have debased a *sacred* literature to the execrable purposes of a sordid ambition.

Biographical memoirs of the living, to be complete, should append an *obituary notice* of the subject. Then curiosity, as well as vanity, would be gratified to the last degree.

How many of those, who are comparatively obscure members of the profession, are devoting untiring energy, and marked ability, in large fields of labor, gathering rich stores of experience, esteemed, respected and beloved by a large circle of friends, and who would be able to flood our medical journals with a simple history of where they were born, whether second or third sons, who their father was, whether born in a LOG house, or a frame house, painted red. Whether nature had endowed them with a spare frame or one capable of great physical power and endurance. Where they received an academic education, or what *M. D.* gave them office instruction. Whether rigid economy compelled them to live on roast potatoes, pudding and milk, &c., &c. Where they were married, when elected delegates to some county medical society, or chairman of a village debating club; and a thousand other trivial incidents in the life of every physician which are common to many others.

Is it not fortunate for our medical literature that the profession, as a whole, are modest enough to permit their "biographical memoirs" to remain unwritten, or at least to rest quietly in their diaries? Otherwise medical biographies of living medical men would vie in number with our yellow covered novels, and *eminent men* would multiply in proportion.

When rightly considered, auto-biography of medical men, or sketches of biographical memoir, published to the world, while the subjects thereof are living, whether they chance to be scientific professors in medical colleges, or modest unassuming country practitioners, is a *gross violation* of Sec. 3d in the Code of Medical Ethics adopted by the National Medical Association, and therefore is as reprehensible and odious as any other can be. In Chapter 2d, Article 1st, we have the following section:

"Sec. 3. It is derogatory to the dignity of the profession to resort to public advertisements, or private cards, or handbills, inviting the attention of individuals affected with particular diseases, publicly offering advice and medicine to the poor, gratis; or promising radical cures; or to publish operations in the daily prints, or to suffer such publications to be made, to invite laymen to be present at operations; to boast of cures and remedies; *to adduce certificates of skill* and success, *or to perform any similar acts.* These are the ordinary practices of empirics, and are highly reprehensible in a regular physician."

In a biographical memoir now before me (in the New Jersey Medical Reporter, for May, 1855,) the subject of which, at latest dates was living, we extract the following:

"As a physician it is no unjust praise to say, that he is universally beloved and esteemed by those who come under his care, inspiring in them confidence and hope, and meeting them with an encouraging smile and a remark oftentimes, more effective for good, than the most potent anodyne. In his diagnosis of disease he is minute, careful and searching: equaled only by the skill and sacred judgment which he exhibits in their treatment. In emergencies requiring self-possession and prompt action, he is calm, deliberate, and efficient, exhibiting a ready command of resources, *which few, under like circumstances, possess.* These attributes have secured for him, in a remarkable degree, the confidence of his professional brothren and of the community in which he lives."

Now whether all this be true, or whether it be exaggerated, are questions altogether irrelevant. This portrait may be a good one, its lights and shades well spread; but in the name of all that is modest and of good report, and the law of medical ethics, is it the proper time to hold it up to the public gaze? Is it not a violation of the spirit and clearly expressed intention of sec. 3d of our orthodox code? Would it not have sounded better if written as a tribute to some veteran of the profession who had "passed away," and whose attributes and characteristics we would love and cherish? On the contrary, the subject of this memoir is still a candidate for public favor and patronage, and, moreover, in all probability approves of that very medical code adopted by the National Medical Association, and denounces even the appearance of quackery.

What better advertisement would an itinerant empiric ask than his biographical memoir, setting forth his claim to public favor and patronage on the ground of his ability to treat diseases *with extraordinary success.*

It is, then, a dangerous precedent, appearing in the garb of dignity commensurate with the character of *true* science: while in reality it is but the vesture of a mountebank.

If medical men are an honor to their profession while living, and are worthy of the immortality of biographical literature, they may rest from their labors in the sleep of *death*, but like Hunter and Cooper, they will "still live." R*******

DETROIT, Oct. 1855.

